# Main reasons for medical consultations in family healthcare units in the city of Recife, Brazil: a cross-sectional study: Principais motivos de consultas médicas em unidades de saúde da família na cidade do Recife, Brasil: um estudo transversal

**DOI:** 10.1590/1516-3180.2014.9490902

**Published:** 2015-08-03

**Authors:** Rinailda de Cascia Santos Torres, Karine Sobral Marques, Kamila de Nazaré Ribas Leal, Pedro Augusto Sampaio Rocha-Filho

**Affiliations:** I Medical Student, Universidade de Pernambuco (UPE), Recife, Pernambuco, Brazil.; II MD, PhD. Adjunct Professor, Department of Neuropsychiatry, Universidade Federal de Pernambuco (UFPE), and Neurologist, Universidade de Pernambuco (UPE), Recife, Pernambuco, Brazil.

**Keywords:** Health services, Primary health care, Community health centers, Epidemiology, Pain, Serviços de saúde, Atenção primária à saúde, Centros comunitários de saúde, Epidemiologia, Dor

## Abstract

**CONTEXT AND OBJECTIVE:**

Only a few studies have focused on the main reasons for consultations at primary healthcare units within the Family Health Program. The aim here was to describe the reasons that led patients to seek assistance at four primary healthcare units in the city of Recife, Brazil.

**DESIGN AND SETTING:**

Cross-sectional study at primary healthcare units in the city of Recife.

**METHODS:**

Among adult patients who were consecutively attended at four primary healthcare units in the city of Recife, their two main reasons for going there were recorded by medical students. The students did not interfere in the consultation dynamics. The data were gathered between September 2010 and March 2011 and between November 2012 and August 2013. The reasons for the consultations were grouped into broader categories in accordance with the International Classification of Primary Care (ICPC-2).

**RESULTS:**

478 patients were included. Their mean age was 45.9 years (± 16 years) and 71% were female. Pain was the main reason for seeking medical attention (34%), followed by evaluation of tests, prescription renewal and medical certificates (17.6%). The most frequent types of pain were musculoskeletal pain (15.7%), headache (10.4%) and abdominal pain (8%). The main reasons for consultation according to ICPC-2 were in the general and nonspecific, musculoskeletal, nervous system-related and digestive tract categories.

**CONCLUSION:**

Pain was the most frequent reason for seeking medical attendance at these primary healthcare units.

## RESUMO

**CONTEXTO E OBJETIVO:**

Existem poucos estudos sobre quais as principais causas de atendimento nas unidades básicas de saúde do Programa de Saúde da Família. O objetivo foi descrever quais as razões que levam os pacientes a procurar quatro Unidades Básicas de Saúde da cidade do Recife, Brasil.

**TIPO DE ESTUDO E LOCAL:**

Estudo transversal em unidades básicas de saúde na cidade do Recife.

**MÉTODOS:**

Pacientes adultos consecutivamente atendidos em quatro unidades básicas de saúde na cidade do Recife tiveram as duas principais razões para o atendimento registradas por estudantes de medicina. Os estudantes não interferiram na dinâmica da consulta. A coleta de dados ocorreu entre setembro de 2010 e março de 2011 e entre novembro de 2012 e agosto de 2013. As razões para as consultas foram agrupadas em categorias mais amplas de acordo com a Classificação Internacional da Atenção Primária (ICPC-2).

**RESULTADOS:**

478 pacientes foram incluídos, a idade média foi de 45,9 anos (± 16 anos), 71% eram do sexo feminino. A dor foi a principal razão para procura de atendimento médico (34%), seguida por avaliação de exames, renovação de receitas ou atestados médicos (17,6%). A dor musculoesquelética foi a mais frequente (15,7%), seguida por cefaleia (10,4%) e dor abdominal (8%). As principais causas de atendimento foram gerais e não específicas, musculoesqueléticas, relacionadas ao sistema nervoso e ao aparelho digestivo (ICPC-2).

**CONCLUSÃO:**

A dor foi o motivo mais frequente para procurar atendimento médico nas unidades básicas de saúde.

## INTRODUCTION

The Family Health Program (Programa de Saúde da Família, PSF) is put into practice through multiprofessional teams that are based at primary care facilities. Recife is the capital of the state of Pernambuco, in the northeastern region of Brazil. PSF was the strategy used by Recife’s Public Health Department to reorganize its primary care provision. Recife is divided in six healthcare districts. District I has nine family health teams; district II, 17 teams; district III, 22 teams; district IV, 32 teams; district V, 15 teams; and district VI, 31 teams. According to data from the Primary Care Information System (SIAB), more than 200,000 families had been registered for attendance through the PSF in Recife up to August 2013.^1^

Only a few studies have focused on the main reasons for seeking consultations at primary care units. The main reasons have been found to relate to general and unspecified, respiratory, digestive and musculoskeletal symptoms.^2^^,^^3^^,^^4^

Knowing what kind of illnesses are treated by PSF teams is extremely important for training the teams and planning primary care, thereby making it possible to create a list of priorities and alternatives for solving these problems.

## OBJECTIVE

The purpose of this article was to describe the most frequent reasons for seeking consultations at four primary care facilities in the city of Recife, Brazil.

## METHODS

This study was carried out from September 2010 to March 2011 and from November 2012 to August 2013 in four primary care facilities in the city of Recife (Santo Amaro, in healthcare district I; Chié, in district II; Alto do Maracanã, in district III; and Sinos, in district IV). These units are linked to Pernambuco University and to the “Professor Fernando Figueira” Integral Medicine Institute, and they were chosen because of their availability of structural conditions for receiving undergraduates.

Patients over the age of 18 who were consecutively attended during the study periods, at medical consultations that had previously been scheduled at the four primary care facilities, were included.

The data were recorded by medical undergraduates who did not interfere in the consultation dynamics and did not ask the patients any questions. The patients’ two main reasons for seeking the consultation were logged through an open questionnaire during the medical consultation, based on what was said to the doctor. The students were trained for three months before the beginning of the study.

All the patients gave their informed consent for their inclusion in the study. The study was approved by the Research Ethics Committees of the “Professor Fernando Figueira” Integral Medicine Institute and of the Oswaldo Cruz University Hospital.

The reasons for seeking medical consultations were grouped into broader categories in accordance with the 17 chapters of the second edition of the International Classification of Primary Care (ICPC-2).^5^

The statistical analysis was carried out using Epi-Info 7.1.3.10 for Windows. All the variables were analyzed descriptively. Means and standard deviations were calculated for quantitative variables. Absolute and relative frequencies were calculated for qualitative variables. Categorical variables were compared across groups using the chi-square test.

## RESULTS

The total number of patients seen at the primary care units over the study period was 481. Three patients refused to participate in the study. Thus, 478 patients were included in the study, of whom 338 (71%) were female. The patients’ mean age was 45.9 years (± 16 years).

Table 1 shows the reasons for seeking consultations at the primary care units. Pain was the most commonly reported complaint at all the primary care facilities.

**Table 1.**
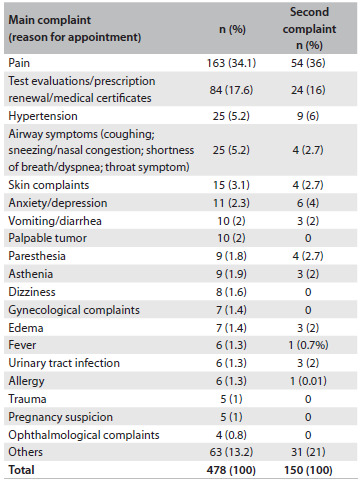
Patients’ most frequent reasons for seeking medical consultations at primary care units

The kinds of pain reported by patients were as follows, in frequency order: musculoskeletal pain (15.7%; 75/478); headache (10.4%; 50/478); abdominal pain (8%; 38/478); and chest pain (1.1%; 5/478). The most frequent types of musculoskeletal pain were back pain (33%) and joint pain (33%).

Table 2 shows the associations between the sociodemographic variables, primary care facilities and reasons for seeking consultations. Older individuals had significantly less pain and asked for more evaluations of tests, prescription renewals and medical certificates. Women had significantly more pain and more reasons for seeking consultations.

**Table 2.**
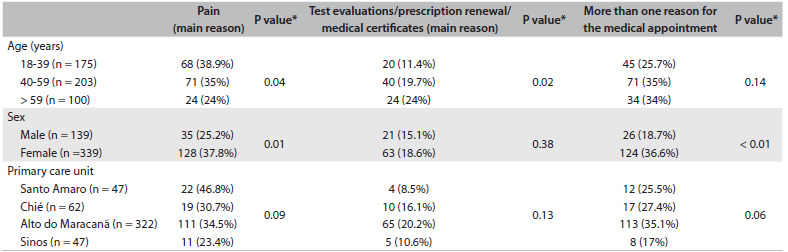
Association of patients’ characteristics and primary care unit with the reasons for seeking the medical consultation

*Chi-square test.

Table 3 shows the reasons for seeking consultations, grouped according to ICPC chapters. The most common reasons were in the categories of the “general and unspecific”, “musculoskeletal”, “neurological” and “digestive” chapters.

**Table 3.**
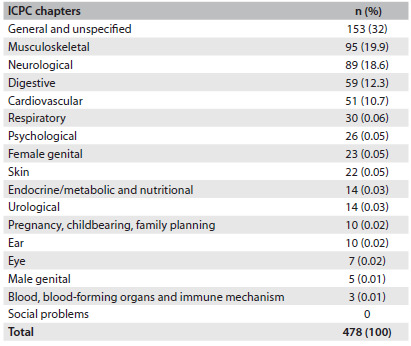
All reasons for seeking medical consultations grouped according to the 17 chapters of the International Classification of Primary Care (ICPC-2)

## DISCUSSION

Pain was the most frequent reason for seeking consultations at primary care units, according to our study. Other authors have also found that pain is an important reason for seeking consultations at primary care units.^2^^,^^4^ Magnago et al. found a different result, but they used a structured questionnaire that did not include pain among its options.^6^

Although pain is a frequent reason why patients seek medical consultations, it is not being properly evaluated and treated.^4^ If primary care teams are trained to diagnose the causes of pain and to provide treatment for this symptom, the demand among patients for healthcare at levels dealing with cases of higher complexity, as well as overloading of emergency services, can be avoided.

A significant number of patients went to the primary care unit to request prescription renewals and have tests evaluated. This was concordant with what was found in other studies.^4^^,^^6^^,^^7^

Even though hypertension had previously been found to be one of the most frequent reasons for consultations, its frequency in our study was lower than what we had expected. With the aim of providing attendance for hypertension and diabetes mellitus patients, a reorganized attendance plan for these diseases, known as HIPERDIA, was created in 2002. This made it possible to register and attend to these patients through primary care.^8^ Attendance for hypertension and diabetes patients at these units follows the program’s enrollment flow, with participation in weekly meetings with multidisciplinary teams. At these meetings, the doctor renews and modifies prescriptions, talks about healthy habits and lifestyles in order to improve clinical control over these illnesses and answers questions. Patients are enrolled in the program after the diagnosis of diabetes or hypertension has been confirmed through an earlier appointment. Through this procedure, patients attended within HIPERDIA only seek medical consultations in cases of complications relating to these diseases.

Women presented significantly more reasons for seeking medical consultations in our study. As far as we know, only one previous study evaluated what is related to the number of reasons for medical consultations within primary care in Brazil. That study also found that women presented more reasons than men did, and that the number of reasons increased with advancing age.^4^

We found that the most common reasons for seeking medical consultation, as categorized in the chapters of the ICPC related to the “general and unspecific”, “musculoskeletal”, “neurological” and “digestive” chapters. These were among the most frequent reasons in another two studies,^3^^,^^4^ and the “general and unspecific” category was the first reason in one of them.^4^

Our study has some limitations. The primary care facilities were selected using convenience criteria and not randomly from among all the units in the city. The units selected are teaching units that regularly receive medicine undergraduates and may not be representative of the city’s primary care. Nevertheless, we tried to include primary care facilities from four of the city’s six healthcare districts. Sixty-seven percent of the patients were interviewed at a single primary care unit. However, it should be highlighted that we did not find any significant differences among the primary care facilities.

## CONCLUSIONS

Pain was the most frequent reason for seeking medical attention at all the primary care facilities, followed by requests for test evaluation, prescription renewal and medical certificates. The most frequent type of pain was musculoskeletal pain, followed by headache and abdominal pain. The most common reasons for seeking medical consultations, according to the ICPC categories related to the general and unspecific, musculoskeletal, neurological and digestive chapters.
